# Assessment of Concentrations of Heavy Metals in Postmyocardial Infarction Patients and Patients Free from Cardiovascular Event

**DOI:** 10.1155/2021/9546358

**Published:** 2021-01-31

**Authors:** Grzegorz Józef Nowicki, Barbara Ślusarska, Andrzej Prystupa, Eliza Blicharska, Agnieszka Adamczuk, Tomasz Czernecki, Krzysztof Jacek Jankowski

**Affiliations:** ^1^Department of Family Medicine and Community Nursing, Medical University of Lublin, Lublin, Poland; ^2^Department of Internal Medicine, Medical University of Lublin, Lublin, Poland; ^3^Department of Analytical Chemistry, Medical University of Lublin, Lublin, Poland; ^4^Department of Physical Chemistry of Porous Materials, Institute of Agrophysics, Polish Academy of Sciences, Lublin, Poland; ^5^Department of Biotechnology, Microbiology and Human Nutrition, University of Life Sciences in Lublin, Lublin, Poland; ^6^Department of Family Medicine, Medical University of Lublin, Lublin, Poland

## Abstract

Cardiovascular diseases (CVDs) constitute the first cause of death among the population of developing and developed countries. Atherosclerosis, which is a disorder with multifactorial etiopathogenesis, underlies most CVDs. The available literature includes ample research studies on the influence of classic cardiovascular (CV) risk factors. However, environmental exposure to heavy metals, among other substances, is still an unappreciated risk factor of CVDs. This study aimed to assess the concentration of some heavy metals (copper (Cu), zinc (Zn), manganese (Mn), cobalt (Co), and iron (Fe)) in the blood serum of postmyocardial infarction (post-MI) patients and patients free from myocardial infarction (MI) as well as estimate the relationship between the occurrence of MI and increased concentration of heavy metals. The concentration of heavy metals (Cu, Zn, Mn, Co, and Fe) was assessed using the inductively coupled plasma mass spectrometry technique in a group of 146 respondents divided into two groups: post-MI group (study group (SG), *n* = 74) and group without cardiovascular event (CVE) having a low CV risk (control group (CG), *n* = 72). The concentration of the analyzed heavy metals was higher in SG. All the heavy metals showed a significant diagnostic value (*p* < 0.001). The highest value of area under the curve (AUC) was observed for manganese (Mn) (0.955; 95% confidence interval (CI) = 0.922–0.988), while the lowest value was found for zinc (Zn) (0.691; 95% CI = 0.599–0.782). In one-dimensional models, high concentrations of each of the analyzed heavy metals significantly increased the chances of having MI from 7-fold (Cu) to 128-fold (Mn). All the models containing a particular metal showed a significant and high discrimination value for MI occurrence (AUC 0.72–0.92). Higher concentrations of Cu, Zn, Mn, Co, and Fe were found to considerably increase the chances of having MI. Considering the increasingly higher environmental exposure to heavy metals in recent times, their concentrations can be distinguished as a potential risk factor of CVDs.

## 1. Introduction

Cardiovascular diseases (CVDs) constitute the main health problems, and hence decreasing the mortality rate and the consequences of these diseases is one of the priority healthcare tasks in developed countries [1]. Atherosclerosis, which is a disease with multifactorial etiopathogenesis, underlies most CVDs [2]. The existing literature includes extensive research on the effect of classic cardiovascular (CV) risk factors such as age, male gender, cigarette smoking, hypercholesterolemia, hypertension, and obesity on CVDs. However, environmental exposure to heavy metals, among other substances, is still an unappreciated CV risk factor [3, 4]. In the last few decades, exposure to heavy metals has become a global public health issue because of its potential health consequences for people [3, 5]. Considerable epidemiological and experimental evidence reveals the influence of lead (Pb) and cadmium (Cd)—metals that are widely present in the environment—on the development of CVDs. A systematic review validated the epidemiological data regarding the impact of Pb and Cd on the development of CVDs [6, 7], while a few animal studies proved that atherosclerosis of the aorta is induced by the metals [8]. Other research showed that essential elements in the blood serum (including Iron (Fe), cobalt (Co), copper (Cu), zinc (Zn), and selenium (Se)) can play a role in the development of CVDs in the general population [9, 10], although the findings are inconsistent. Some evidence suggests that even low concentrations of heavy metals such as arsenic (As) and cadmium (Cd) in the environment can adversely affect human health [11].

Heavy metals, frequently defined as those with a density higher than 5 g/cm^3^, such as Cu, Zn, Mn, Co, and Fe, are widespread in the Earth's crust, but they exist in low concentrations in living organisms. Even small traces of such metals in the atmosphere, soil, and water can cause serious health problems in the organisms. The main influence of heavy metals on people's health results primarily from occupational exposure, environmental pollution, or their accumulation in food [12]. The mechanism underlying the influence of heavy metals on the risk of CVDs has not been thoroughly studied, although many documents have demonstrated their immunotoxic and carcinogenic effects [13]. The potential relationship between exposure to heavy metals and the occurrence of CVDs can be associated with oxidative/antioxidative imbalance in the organisms [14]. The pathogenesis of CVDs caused by exposure to heavy metals can involve the production of a large amount of free radicals capable of destroying DNA or inducing lipid peroxidation and depletion of protein sulfhydryls (e.g., glutathione) [15].

Scientific evidence indicates both positive and negative influences of heavy metal concentrations on the CV risk in humans [6, 16]. Because the human body is exposed to increasingly higher concentrations of heavy metals in the environment, this study aimed to assess the concentration of selected heavy metals (Cu, Zn, Mn, Co, and Fe) in the blood serum of postmyocardial infarction (post-MI) patients and patients free from myocardial infarction (MI) as well as determine the relationship between MI occurrence and higher concentrations of these metals. The following hypothesis was formulated: higher concentrations of the analyzed heavy metals (Cu, Zn, Mn, Co, and Fe) are associated with a greater probability of MI occurrence.

## 2. Materials and Methods

### 2.1. Study Design and Participants

The study was designed to have an 80% chance of detecting a 0.46 standardized mean difference in Cu concentration using a *p* value of 0.05, based on the values given in a published article [17]. To demonstrate a similar or greater difference, 60 patients or more were required in each group. However, as a nonparametric test had to be used, 15% of additional subjects were added to each group [18]. This cross-sectional study was conducted from August to December 2017 among 146 adults divided into two groups: the study group (SG) and the control group (CG).

The SG constituted patients with a post-MI condition, who were hospitalized in the early period of cardiac rehabilitation (up to the 14^th^ day following their discharge from hospital after complete revascularization) and continued physiotherapy, diet therapy, and pharmacological therapy in the therapeutic unit “Uzdrowisko Nałęczów” S.A. in Nałęczów and the Railway Health Resort Hospital in Nałęczów (Eastern Poland). The research included patients from consecutive rehabilitation stays who were held every 21 or 28 days. All the SG patients had experienced MI once and were treated using percutaneous coronary intervention (PCI). The inclusion criteria were as follows: age between 40 and 65 years, post-MI condition, and written informed consent to take part in the study. The exclusion criteria were the following: a medical history of renal failure, neoplasm, lung disorder, or rheumatic disease, and age below 40 years or above 65 years. Another exclusion criterion encompassed the consumption of dietary supplements containing trace elements and vitamin complexes, which could affect the level of heavy metals in the blood serum.

The CG constituted adults who had never had cardiovascular event (CVE) and attended routine checkups with an occupational medicine physician during periodic examinations. The respondents were recruited from the Regional Preventive and Therapeutic Centre of Occupational Medicine in Lublin (Eastern Poland). The inclusion criteria were as follows: age between 40 and 65 years, absence of CVE in medical history, low 10-year CVE risk (SCORE < 5) [19], lack of chronic diseases (renal failure, neoplasm, rheumatic disease, and lung disorder), lack of CV complaints suggesting CVD based on atherosclerosis, no consumption of dietary supplements containing trace elements, lack of hypertension therapy, and lack of prediabetes and hypercholesterolemia. The exclusion criteria included presence of an active infection and consumption of drugs or dietary supplements that could affect the level of heavy metals in the blood serum.

The research project was approved by the Bioethics Commission at the Medical University of Lublin (KE-0254/197/2017) and was conducted in accordance with the Declaration of Helsinki. All the respondents were informed about the aim of the study and provided a written informed consent to participate in the research.

### 2.2. Blood Sample

Blood samples were collected from the ulnar vein of patients, while fasting in the morning (7–9 a.m.) after a full night's rest. The samples were taken in a tube coated with a clot activator and separating granules and then delivered within an hour to a laboratory. The tubes containing samples were stored at 4°C until delivered. Plasma was separated from the samples by centrifuging at a speed of 3 000 revolutions per minute for 10 minutes. The serum was centrifuged and directly placed in Eppendorf tubes and then stored at −80°C to check the concentrations of Cu, Zn, Mn, Co, and Fe.

### 2.3. Metal Biomarker Levels

#### 2.3.1. Chemicals

Suprapur grade nitric acid (65% HNO_3_) was purchased from Merck (Germany). Ultrapure water (Milli-Q, Millipore, Raleigh, NC, USA, resistivity 18.2 MΩ cm) was used for preparing 1% HNO_3_ and for diluting the samples. Additionally, the certified single-element standard solutions of Cu, Mn, Co, Zn, and Fe (each at a concentration of 1000 mg/l, purity grade 99.99%) were obtained from Agilent Technologies (USA). The certified reference material ClinChek® Plasma Control for Trace Elements, Level I (Germany), was prepared according to the manufacturer's instructions.

#### 2.3.2. Preparation of Samples

Human plasma samples (0.2 ml each) were collected, and the organic matter was digested by adding 2 ml of 65% HNO_3_ (Merck, Darmstadt, Germany). Wet mineralization was conducted by using a Mars 6 microwave mineralization system (CEM, Matthews, NC, USA). After adding 2 ml of 65% HNO_3_ to samples, they were transferred to Teflon containers and digested at 180°C. Later, the samples were diluted by adding 5 ml of ultrapure water and analyzed to determine the concentration of the respective trace elements.

#### 2.3.3. ICP-MS Analysis

The concentrations of Cu, Mn, Co, Zn, and Fe in the diluted samples were determined using inductively coupled plasma mass spectrometry (ICP-MS) (820-MS, Varian, Australia). The following parameters were used during the ICP-MS analysis: plasma gas flow 18 l/min, nebulizer gas flow 1 l/min, frequency power 1.37 kW, and auxiliary gas flow 1.70 l/min. To prepare the calibration curve (corresponding to the concentrations of trace elements in human plasma), the certified single-element standards were mixed and diluted in 1% HNO_3_. The human plasma samples and quality control samples were analyzed in triplicate, and the average values were used as the final results.

### 2.4. Other Variables

The medical interview, physical examination, and sample collection were performed by a well-trained nurse. Sociodemographic data (age, gender, place of residence, education, and assessment of smoking status) were obtained using standard questionnaires. A respondent who had smoked at least one cigarette per month was considered as a smoker [20]. All the respondents underwent anthropometric tests to determine their height and body weight. Height was measured on a stadiometer with an accuracy of up to 0.1 cm, while weight was checked without shoes and outer clothes using platform scales with an accuracy of up to 0.1 kg. The body mass index (BMI) of all the respondents was defined as body weight (kg) divided by height (m) squared(kg/m^2^) [21].

### 2.5. Statistical Analysis

The distribution of variables was presented as follows: numbers and percentages for categorical variables, means with standard deviations (SDs) for normally distributed continuous variables, and medians with interquartile ranges for continuous variables with skewed distribution. The Kolmogorov–Smirnov test was used to verify the normality of the data distribution. The SG and CG were compared using Student's *t*-test or Mann–Whitney *U* test when considering quantitative variables and using Chi-square test when considering qualitative variables. The diagnostic usefulness of specific heavy metals for detecting MI was calculated using receiver operating characteristic (ROC) curves with area under the curve (AUC). A cut-off value corresponding to the highest accuracy rate was determined, and the related sensitivities and specificities were summarized in the Youden index. The multivariable logistic regression model was used to investigate the association between CVE and specific heavy metals (based on the cut-off value determined from the Youden index). The results of the logistic regression model were presented as odds ratios (ORs) and 95% confidence intervals (CIs). The ROC curves were used to assess the discrimination of a fitted logistic model. Moreover, the AUC values for ROC curves with 95% CIs were calculated. All the analyses were performed in IBM Corp. Released 2017. IBM SPSS Statistics for Windows, Version 25.0. Armonk, NY: IBM Corp. Statistical significance was defined as *p* < 0.05.

## 3. Results

### 3.1. Characteristics of the Analyzed Group

Table 1 presents the descriptive statistics of the studied groups. All participants lived on Polish territory and were Caucasians. In the final analysis, 146 respondents were included, out of which 74 (51%) were in the SG. Mean age was higher in the SG (57.38 (SD = 4.72) years) than in the CG (53.67 (SD = 6.32) years). No significant differences were found in the distribution of gender between the groups (*p*=0.13). The CG contained a higher percentage of individuals living in urban areas with higher education and a lower percentage of smokers than the SG. A significant difference was observed in the distribution of each heavy metal between the SG and the CG. The concentrations of the analyzed heavy metals were higher in the SG.

### 3.2. ROC Curve and Cut-Off Value for the Analyzed Metals

**Figure 1:**
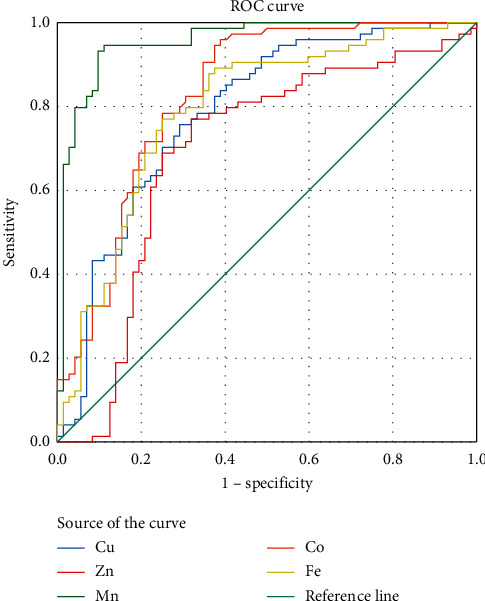
ROC curve analysis of the analyzed plasma metals for diagnosing MI.

Table 2 and Figure 1 present the results of the ROC analysis with AUC and the cut-off value determined based on the Youden Index for the five heavy metals (Cu, Zn, Mn, Co, and Fe). All the heavy metals showed significant utility for diagnostic MI (*p* < 0.001). The highest value of AUC was observed for Mn (0.955; 95% CI = 0.922–0.988), while the lowest value was observed for Zn (0.691; 95% CI = 0.599–0.782). The cut-off values determined for heavy metals were slightly lower than their median values for the sample. The heavy metal Co displayed the highest percentage of distribution exceeding the cut-off value in 96 respondents (65.8%).

### 3.3. Analysis of the Relationship between Prevalence of MI and Higher Concentrations of the Analyzed Heavy Metals

Table 3 presents the results for the analysis of relationship between the prevalence of MI and higher concentrations of the heavy metals (estimated by the ROC analysis) for the four models of logistic regression, after adjusting for potential confounders. Model 1 includes a heavy metal as an independent variable. Model 2 is adjusted for age and gender. Model 3 additionally takes into account the influence of smoking status. Model 4, the complete model, includes obesity, education, and place of residence along with the abovementioned variables. In simple models, high concentrations of each metal significantly increased the probability of the prevalence of MI from 7-fold in the case of Cu to 128-fold in the case of Mn. All the models containing a particular metal (model 1) showed significant and high discrimination values for the prevalence of MI (AUC 0.72–0.92). For Cu, Zn, and Fe, after adjusting for age, gender, smoking status, obesity, education, and place of residence, the direction and strength of the estimated association between the analyzed metals and MI did not change considerably. However, an increase in the discrimination values of the models was found: the highest for Zn, 22% and the lowest for Cu, and Fe, 18% and 17%, respectively. For Mn and Co, after adjusting for gender, age, and smoking status (Model 3), a meaningful increase in OR was observed. The size of the estimated effect was maintained in Model 4. The smallest increase in the discrimination value of the models was found for Mn, from 0.92 (Model 1) to 0.96 (Model 4).

## 4. Discussion

In this cross-sectional study, concentrations of heavy metals (Cu, Zn, Mn, Co, and Fe) were compared in the SG and CG and the relationship between MI occurrence and higher concentrations of the metals was estimated. To recapitulate, the results indicated that concentrations of the analyzed heavy metals were significantly higher in the blood serum in the group of individuals who had MI in their medical history. Moreover, higher concentration of each heavy metal (determined using the ROC curves) was shown to considerably increase the probability of being diagnosed with MI. The highest discrimination value of the model determining the relationship between MI and the selected heavy metals was found for Mn. As far as known, the harmful influence of heavy metals on the CV system has not been studied thoroughly [6]. The potential proatherogenic effect, even if it is minor as compared to traditional risk factors (i.e., smoking, hypertension, and obesity) would have some significance, especially in a population exposed to heavy metals, for example, due to the type of profession [14]. The main sources of environmental pollution with heavy metals are industry, traffic, smoking, and food [22]. It is well known that Cu, Zn, Mn, Co, and Fe are essential trace elements present in humans. Absorption and excretion of these elements should be maintained at the desired level because lack or excess of these metals in the body can be harmful to human health.

As a strong prooxidant, Cu may play a role in the development of atherosclerosis, which underlies cardiovascular diseases [23]. Similarly to the present study, the meta-analysis by Chan et al. [24] indicated that a higher concentration of Cu was found in the blood serum of post-MI individuals as compared to those in the healthy group. Altekin et al. [25] noticed a positive correlation between the level of Cu in the serum and the markers of myocardial damage, including cardiac troponin T and troponin I, and mass concentration of creatine kinase isoenzyme MB. Kazi et al. [26] performed research on a group of 130 post-MI patients and healthy adults aged 45–60 years. The post-MI patients were divided into three subgroups depending on the number of MIs they had had: those with one, two, or three MIs. As in the present study, the authors detected that concentrations of Fe and Cu in the blood serum were higher in patients after 1–3 MIs than in the control group participants. However, in contrast to the present study's findings, the concentration of Zn was higher in the post-MI patients in all the three subgroups as compared to those in the control group. Spasojevic-Kalimanovska et al. [27] proved that the risk factors of coronary heart disease (inflammatory factors, obesity, higher lipid concentrations, oxidative/antioxidative status, etc.) were associated with a higher concentration of Fe, which indicates that Fe status can be useful in the prediction of CVDs, while Domingo-Rellose et al. [28] concluded that exposure to Cu and Zn was associated with an increased risk of CVD. Improper zinc homeostasis is associated with the development of cardiovascular diseases [29]. Liu et al. [30] conducted a meta-analysis aimed at assessing the relationship between Zn concentration in the body and the occurrence of MI. The results of 13 publications proved that patients with acute MI had lower Zn concentration than the control group. This is in contrast to the current study, in which Zn concentration was higher in the SG than in the CG. This may be due to decrease of the level of Zn decreases in patients with acute MI due to its absorption into undamaged cells of the myocardium where it is used in cardiac repair processes [31]. Furthermore, Jain and Mohan [32] showed that Zn level in the blood serum of post-MI patients decreased within the first 24 hours until the fourth day and then increased to the normal value on the14^th^day from the incidence of MI. Similarly, Low and Ikram [33] stated that, in post-MI patients, the level of Zn in the blood serum decreased within the first 3 days of the occurrence of MI and then increased up to the normal level until the 10^th^ day, which shows that a low level of Zn in the blood serum can be an effect and not a cause of CVDs. However, research on the influence of Zn ions on the pathology of the CV system is still insufficient, and many key issues remain unexplained.

Mn exposure is associated with a higher risk of hypertension and mortality from CVD [34]. In the current study, the level of Mn in the blood serum of the SG patients was higher compared to the CG. Moreover, in one-dimensional models, a high concentration of Mn was observed to considerably increase the risk of MI. Afridi et al. [35] assessed Cr and Mn concentrations in the population of patients with acute MI in Pakistan and reported contrary results to those of the present study. Consistent with their findings, the post-MI patients (following one, two, or three MIs) examined in this study had a lower level of Mn in the blood serum than the CG participants. However, Ilyas and Shah [36] found a higher Mn concentration in the blood serum of patients with coronary artery disease (CAD) compared to the control group. Yuan et al. [37] and Zhu et al. [38] did not find any association between exposure to Mn and 10-year CV risk. However, in epidemiological studies, a higher level of Mn was found in the blood serum of atherosclerosis patients aged 30–62 years and 61–100 years in comparison with individuals free from changes in blood vessels [39]. Cebi et al. [17] angiographically assessed Mn concentration in the blood serum of CAD patients and in the healthy control group participants. The authors found a higher Mn concentration in CAD patients, which is in line with the findings of the current study. Nevertheless, there is insufficient research explaining how exposure to Mn contributes to damage in the CV system, in both humans and animals [40].

Agarwal et al. [41] observed a significant relationship between Co concentration in the urine and CV risk, while Ari et al. [42] showed a positive correlation between Co concentration in the blood serum and symptoms of subclinical atherosclerosis in hemodialysis patients. The relationship between smoking and CV risk was partially mediated by Co concentration. However, due to limited research assessing the association between Co concentration and CV risk, a hypothesis similar to the one proposed by Wen et al. [43] was formulated: higher Co concentration can influence the development of CVDs.

The hemostasis of essential heavy metals is carefully regulated by a system of protein transporters involved in the uptake, distribution, storage, and excretion of metal ions in the body [44]. In eukaryotes, the vacuolar system also plays a vital role in the hemostasis of metal ions as it collects and transports them across cell membranes via the secretory pathway. In the cell, organelles such as the peroxisome, chloroplasts, and mitochondria serve as reservoirs of metal ions and contribute to their overall hemostasis by using their transport and storage systems [45, 46]. Cu is regulated by several transporter proteins, including copper transporters 1 and 2 (CTR1 and CTR2), Atox1, copper chaperone for superoxide dismutase (CCS), Cox1, Ceruloplasmin, copper transporting ATPase beta (ATP7B), and metallothionein. Fe hemostasis is regulated by transferrin, ceruloplasmin, hephaestin, ferroportin , and the divalent metal transporter 1 (DMT1). Zn ions' concentration is controlled by the combined activity of the different families of zinc transporters (ZnT and ZIP) and metallothionein [47, 48]. The maintenance of Mn hemostasis involves a complex network of proteins that mediate the import or export of Mn, including the divalent metal transporter 1 (DMT1), zinc transporters ZIP8 and ZIP14, the citrate transporter, the choline transporter, the dopamine transporter (DAT), the transferrin receptor (TfR), calcium channels, ATPase 13A2 SLC30A10, ferroportin, and the Ca^2+^ secretory pathway—ATPase 1 (SPCA1) [49, 50]. The main uptake mechanisms identified include DMT1 and Tf-TfR mediated endocytosis. It is worth noting that none of these transporters is specific to Mn as they also transport other metals.

Several potential mechanisms explain the relationship between heavy metal concentration and CVE occurrence. Copper works as a cofactor for different proteins and enzymes necessary for the maturity of cytoplasmic cuproproteins and production of enzymes in different organelles [51]. Appropriate Cu consumption assures protection against Pb, while a higher Cu consumption leads to higher Pb absorption [52], which greatly increases the highly reactive forms of oxygen (e.g., hydroxyl radicals) capable of damaging cells, especially DNA, and inducing oxidation of proteins and lipids [53]. Copper-mediated lipid peroxidation process was demonstrated in several *in vivo* and *in vitro* studies [54]. Formation of Cu complexes with homocysteine is another potential mechanism causing endothelial dysfunction and blood vessel damage [55]. In addition to serving as a catalyst as well as a structural and regulator ion [56], the heavy metal Zn is well known for its role as a cofactor of superoxide dismutase (SOD), a protector of biological structures against damage to free radicals, and an agent that prevents interaction between chemical groups and Fe [57]. Lack of Zn was connected with increased lipid peroxidation in the mitochondrial and microsomal membranes [58]. Apart from the positive effects, Zn supplementation was stated to be associated with the displacement of other metals, thereby decreasing their concentration in the organism [59] (e.g., Cu [60]), and decreasing the levels of high-density lipoprotein (HDL) cholesterol in plasma [61]. The metal Mn plays an essential role as a cofactor of different enzymes participating in the metabolism of amino acids, lipids, and carbohydrates. It works as a potential antioxidant because it is a part of Mn-SOD which protects the cells against damage. Moreover, the finding of relationship between decreased Mn-SOD activity and atherosclerosis suggested that the analysis of Mn contents in the matrix of blood vessel walls can be used in future as one of the diagnostic methods for atherosclerosis at the early stages [62]. Apart from a potentially favorable biological activity, excessive exposure to Mn can cause a neurotoxic effect [63]. Mn can influence blood pressure by decreasing the sensitivity of blood vessels to the activation of alpha-adrenergic receptors, decreasing the level of dopamine, and inducing oxidative stress. It can also antagonize the alpha-adrenergic receptor in blood vessels and calcium channel, thereby affecting the functioning of the autonomic nervous system [64]. As Bae et al. [65] observed in their research on rats with insufficient calcium and ovaries removed, an increase in serum cholesterol level caused by the removal of ovaries and a calcium-insufficient diet can be decreased by Mn supplementation. On the other hand, the level of CV risk can increase by Mn supplementation because a reduction in HDL cholesterol range becomes greater than a reduction in total cholesterol and low-density lipoprotein (LDL) cholesterol. Cobalt is present in the reactive centers of several enzymes (i.e., methylmalonyl-CoA mutase and methionine synthase) in humans. The main function of Co in the organism is to serve as a cobalamin ingredient that regulates erythrocyte production and so its insufficiency leads to anemia [66]. In biological systems, Co (II) complexes produce oxygen radicals that affect the CV, hematologic, nervous, hormonal, and reproductive systems [67]. Iron, in the form of hemoprotein and iron–sulfur centers, is the most common metal in the organism. It significantly contributes to cellular reactions via great elasticity and serves as a donor and recipient of electrons. However, the properties that make it a necessary element also contribute to its potential toxicity via catalyzing reactions that generate free radicals. Hepcidin, an essential hormone synthesized by the liver, regulates the level of Fe in the organism, maintains intracellular availability, and decreases Fe absorption in the intestines [68]. Its activity depends on its binding with ferroportin, a transmembrane protein, which transports Fe from the cell's interior to its exterior. This binding decreases the flow of Fe by an enterocyte, increases Fe confinement in macrophages, and finally leads to atherosclerosis [69]. Moreover, Chaudhary et al. [70] found that Fe overload significantly increased retinal renin expression in mice via succinate receptor signaling, which resulted in neurodegeneration and vascular abnormalities. Therefore, insufficient Fe can prevent the progress of atherosclerotic plaque destabilization in blood vessels [71]. However, the most feasible mechanism through which Fe can contribute to atherosclerotic plaque formation is based on its well-known ability to catalyze the production of reactive oxygen forms, lipid peroxidation, and LDL oxidation [72].

Metal-binding proteins also play an important role in reducing oxidative stress, a factor that directly influences the formation of atherosclerotic lesions in blood vessels. For example, one of MT's important biological roles is protection against oxidative stress induced by various environmental stressors, including toxic metals. In mice studies, knockout mice lacking MT expression were shown to be more sensitive to Cd toxicity than mice in the control group [73]. In general, the large number of –SH groups in the MT molecule allows it to react with numerous electrophilic chemicals because it scavenges free radicals, such as hydroxyl, superoxide, and nitric oxide radicals, formed during the metabolism of xenobiotics [74].

### 4.1. Strengths and Limitations

The strengths and limitations of this study are worth considering. An extensive analysis of the literature suggests that this is the first study where the ROC curves have been applied to define cut-off value for the analyzed heavy metals to diagnose MI. Another strength of this research is that the cut-off value obtained for the analyzed plasma metals were used in the models of logistic regression that enables the determination of significant predictors of MI. Additionally, for each heavy metal, the discriminating value of the model was given, which allows to compare the diagnostic usefulness of heavy metals among them. In this study, the MI diagnosis of the SG patients was confirmed in the medical documents and endorsed by a doctor during an angioplasty procedure. Furthermore, a complex clinical examination was performed on the CG participants to minimize the possibility of having undiagnosed and preclinical CVD.

Nevertheless, this study has several limitations. Firstly, it is a cross-sectional study and thus does not show cause and effect or time and effect associations between the concentrations of heavy metals and the risk of MI. Secondly, only basic sociodemographic data were taken into account (i.e., gender, age, place of residence, and education) along with two lifestyle variables that affect the level of heavy metals (smoking and obesity). A detailed analysis of heavy metal exposure, lifestyle behaviors, and other factors required to thoroughly assess the sources of exposure has not been performed. Thirdly, as each metal has its own placement in the organs and CV system, one specific approach for determining the general status of many metals has not been found. The assessment of plasma concentration cannot be the best way of showing the total concentration of some metals in the organism. However, Yuan et al. [37] analyzed the correlations between the concentrations of some heavy metals in the plasma samples, complete blood samples, and urine samples and, similar to the present study, found comparable levels of concentrations of heavy metals. Fourthly, in this study, the concentrations of the plasma metals were only determined once in the SG and CG and, in the SG, the examination was performed up to two weeks after MI. There is very little information regarding the biological half-life of many metals. Nevertheless, the analysis carried out by Yuan et al. [37] comparing the concentrations of metals in a 5-year interval (in 2008 and 2013) in the population of 138 individuals showed good regeneration of Zn, Mn, and Fe in the serum. While assessing the concentrations of Co and Cr ions in the blood serum of patients before and after the removal of the metal-on-metal hip prosthesis, Durrani et al. [72] found that the change in ion concentrations differed considerably between patients and a higher level of Co and Cr ions can be maintained for up to one year after revision. Lastly, in this study, the coefficients estimated for determining the relationship between high plasma metal concentrations and MI occurrence are very high, associated with a wide range, especially for Mn. These results have been obtained from a relatively small sample and low number of SG participants with a low Mn concentration and CG participants with a high Mn concentration, but the direction and power of the dependencies remained stable which is worth highlighting.

### 4.2. Implications and Future Research

The findings of this study can have important political and scientific implications. The results highlight the significance of heavy metals in the current environment as an independent risk factor increasing the occurrence of MI, apart from traditional risk factors such as hypertension, low physical activity, and inappropriate diet. This might affect the prevention strategy used for no communicable diseases, which have so far focused on the factors determined by lifestyle, for instance, as indicated in the 2008 report of the World Health Organization (WHO) [75]. The recognition of environmental factors, such as high concentrations of heavy metals, as an additional priority in the eradication of diseases of affluence, including CVDs, can gain social and political support for special legislation and proposal of strategies and preventive standards to eliminate the environmental factors that significantly affect the CV health.

Although the findings of this study require validation, they can have important implications for public health, taking into account the high rate of mortality due to CVDs. Further research is required to explain the mechanisms underlying the influence of heavy metals concentration on the risk of CVDs. Such research should be perspective and cohort-based, while taking into account a greater number of patients, and should involve long-term assessment of heavy metal exposure. Thus, future studies should perform repeated measurements of heavy metal concentration during the lifetime of an individual, as this can provide valuable information on the impact of long-term exposure to higher concentrations of heavy metals in the environment on the risk of CVDs. Moreover, this might allow understanding the regeneration of heavy metals. Secondly, future research should take into account the levels of metal-binding proteins, which indirectly reflect the levels of heavy metals in the human body.

## 5. Conclusions

To summarize, this research revealed higher concentrations of Cu, Zn, Mn, Co, and Fe in the blood serum of post-MI patients in comparison with that of individuals free from CVE. Moreover, higher concentrations of the analyzed heavy metals were found to significantly increase the occurrence of MI. Considering the increasingly higher exposure to heavy metals in the environment, this study has numerous implications for public health.

## Figures and Tables

**Table 1:** Characteristics of the analyzed group.

Variables	Study group (*n* = 74)	Control group (*n* = 72)	*p*
*Demographic data*			
Age (years)^b^	57.38 ± 4.72	53.67 ± 6.32	<0.001
Gender (male)^a^	54 (73.0)	44 (61.1)	0.13
Place of residence (city)^a^	31 (41.9)	49 (68.1)	0.001
University education^a^	14 (18.9)	43 (59.7)	<0.001
Smoking^a^	22 (29.73)	7 (9.72)	0.002
Obesity (BMI ≥ 30)	27 (36.5)	13 (18.1)	0.01
*Metals in blood serum*			
Cu (10^−6^) *μ*g/ml^c^	0.97 (0.83–1.13)	0.72 (0.62–0.87)	<0.001
Zn (10^−6^) *μ*g/ml^c^	0.45 (0.40–0.55)	0.36 (0.33–0.42)	<0.001
Mn (10^−9^) ng/ml^c^	2.42 (1.17–4.55)	0.27 (0.12–0.54)	<0.001
Co (10^−9^) ng/ml^c^	0.33 (0.26–0.43)	0.17 (0.12–0.26)	<0.001
Fe *μ*g/ml^c^	0.78 (0.66–1.02)	0.51 (0.38–0.66)	<0.001

Data presented as ^a^*n* (%), ^b^mean ± SD, or ^c^median (Q1–Q3); BMI—body mass index.

**Table 2:** AUC, cut-off value, sensitivity, and specificity for the analyzed heavy metals.

Parameter	AUC	95% CI		*p*	Cut-off value	Sensitivity	Specificity	Youden index
Cu	0.785	0.710	0.861	<0.001	0.840	0.76	0.71	0.47
Zn	0.691	0.599	0.782	<0.001	0.398	0.78	0.68	0.45
Mn	0.955	0.922	0.988	<0.001	0.715	0.93	0.90	0.84
Co	0.828	0.759	0.896	<0.001	0.194	0.95	0.63	0.57
Fe	0.792	0.717	0.867	<0.001	0.655	0.77	0.75	0.52

Note: AUC—area under the curve; CI—confidence interval.

**Table 3:** Relationship between cardiovascular events and higher concentrations of plasma metals.

	Model 1			Model 2			Model 3			Model 4		
	OR	95% CI	AUC (95% CI)	OR	95% CI	AUC (95% CI)	OR	95% CI	AUC (95% CI)	OR	95% CI	AUC (95% CI)
Cu	7	(3.39–15.56)	0.73 (064–0.81)	7.63	(3.51–17.38)	0.80 (0.73–0.87)	8.16	(3.51–18.96)	0.84 (0.77–0.90)	7.22	(2.92–17.87)	0.86 (0.80–0.92)
Zn	7.14	(3.43–14.88)	0.72 (0.63–0.80)	7.14	(3.43–14.88)	0.79 (0.72–0.87)	9.49	(3.87–23.27)	0.83 (0.77–0.90)	9.03	(3.39–24.10)	0.88 (0.83–0.94)
Mn	128.1	(38.7–424.0)	0.92 (0.87–0.97)	128.14	(38.73–424.00)	0.95 (0.92–0.989)	198.7	(43.21–913.7)	0.96 (0.93–0.992)	167.9	(32.9–857.0)	0.96 (0.93–0.99)
Co	23	(8.2–64.1)	0.78 (0.70–0.86)	23	(8.25–64.13)	0.89 (0.84–0.95)	44.4	(12.99–151.86)	0.90 (0.85–0.95)	54.9	(13.54–221.98)	0.93 (0.89–0.97)
Fe	10.1	(4.7–21.51)	0.76 (0.68–0.84)	10.10	(4.70–21.51)	0.85 (0.78–0.91)	11.230	(4.67–27.01)	0.85 (0.80–7.54)	9.84	(3.80–25.45)	0.89 (0.84–0.95)

Model 1: simple linear regression; Model 2: after adjusting for age and sex; Model 3: after adjusting for age, sex, and smoking status; Model 4: after adjusting for age, sex, smoking status, obesity, education, and place of residence; OR—odds ratio; CI—confidence interval; AUC—area under the curve.

## Data Availability

The data used to support the findings of this study are available from the corresponding author upon request.
